# Supplementary material for: Elevation-dependent pattern of net CO2 uptake across China
Source: Nat Commun. 2024 Mar 20;15:2489. doi: 10.1038/s41467-024-46930-4 (PMC10954722; doi:10.1038/s41467-024-46930-4)
Supplement: Supplementary file 1 — Supplementary Information [file 41467_2024_46930_MOESM1_ESM.pdf]

Supplementary Information for

Elevation-dependent pattern of net CO_2 uptake across China

Da Wei [1, 2 #] *, Jing Tao [1, 2 #], Zhuangzhuang Wang [1, 2], Hui Zhao [1], Wei Zhao [1], Xiaodan Wang [1, 3] *

[1] *Institute of Mountain Hazards and Environment, Chinese Academy of Sciences, Chengdu, China;*

[2] *University of Chinese Academy of Sciences, Beijing, China;*

[3] *Institute of Tibetan Plateau Research, Chinese Academy of Sciences, Beijing, China;*

[#] *Equal contribution of these authors*

***Corresponding Authors:**

Prof. Da Wei, weida@imde.ac.cn

Prof. Xiaodan Wang, wxd@imde.ac.cn

Contents: Text S1; Figure S1-S15; Table S1-S3.

Text S1 Representativeness of the eddy covariance dataset

The 203 eddy covariance sites (526 site-years) are a good representation of the major ecosystem types and climate zones in China **(Fig. S1; Table S1)**. The dataset provides enough information about the elevation gradient of mountainous China, with 88 sites at >1000 m altitude. Among these sites, 53 sites are above 2000 m, 42 sites are above 3000 m, 16 sites are above 4000 m and one site is above 5000 m **(Fig. S2)**. Among the terrestrial ecosystem types during 2002 to 2020 (with a mean of 2012), the cropland has the strongest net ecosystem productivity (NEP) (418.4±72.5 g C m^{-2} yr^{-1}, *n*=40; note grain yield is not considered in the eddy covariance measurements), followed by forest (natural forest 376.3±52.2 g C m^{-2} yr^{-1}, *n*=34; planted forest 328.8±66.9 g C m^{-2} yr^{-1}, *n*=19; bamboo −101.4±248.6 g C m^{-2} yr^{-1}, *n*=5), marshland (227.0±47.5 g C m^{-2} yr^{-1}, *n*=39), grassland (83.7±17.0 g C m^{-2} yr^{-1}, *n*=43), shrubland (62.1±17.1 g C m^{-2} yr^{-1}, *n*=10) and desert (41.3±31.9 g C m^{-2} yr^{-1}, *n*=11) **(Fig. S3)**.

The NEP by the different terrestrial ecosystems are generally consistent with the variation in soil organic carbon during the last two decades, as shown by national-scale repeated soil carbon surveys [6] of, for example, forest [7], grassland [8] and cropland [9]. Considering offsite C transport, the largest net CO_2 sink is by forests (note most C in croplands is consumed by humans, resulting almost a neutral CO_2 sink in croplands [10]), consistent with several previous studies [11-13]. For natural forest, the NEP in EddyChina2023 is similar to that in a previous report [12]. Planted forest takes up CO_2 at a rate similar to natural forest, which may largely be due to their younger stand age of roughly 30 years [14]. A negative relationship has been reported between the NEP and stand age across China's planted forests [12]. For grassland, the EddyChina2023 dataset suggests that the temperate grasslands of Inner Mongolia are not significantly different from neutral, consistent with repeated soil carbon inventory results [15], largely due to increasing drought. By contrast, the alpine grasslands of the Tibetan Plateau, which have a cold, dry climate, function as a net CO_2 sink under a warming and wetting climate [16], in line with repeated soil sampling [8]. These analyses validate our eddy covariance dataset in terms of the representativeness of China's terrestrial net CO_2 uptake, enabling further analyses of these mountainous regions.

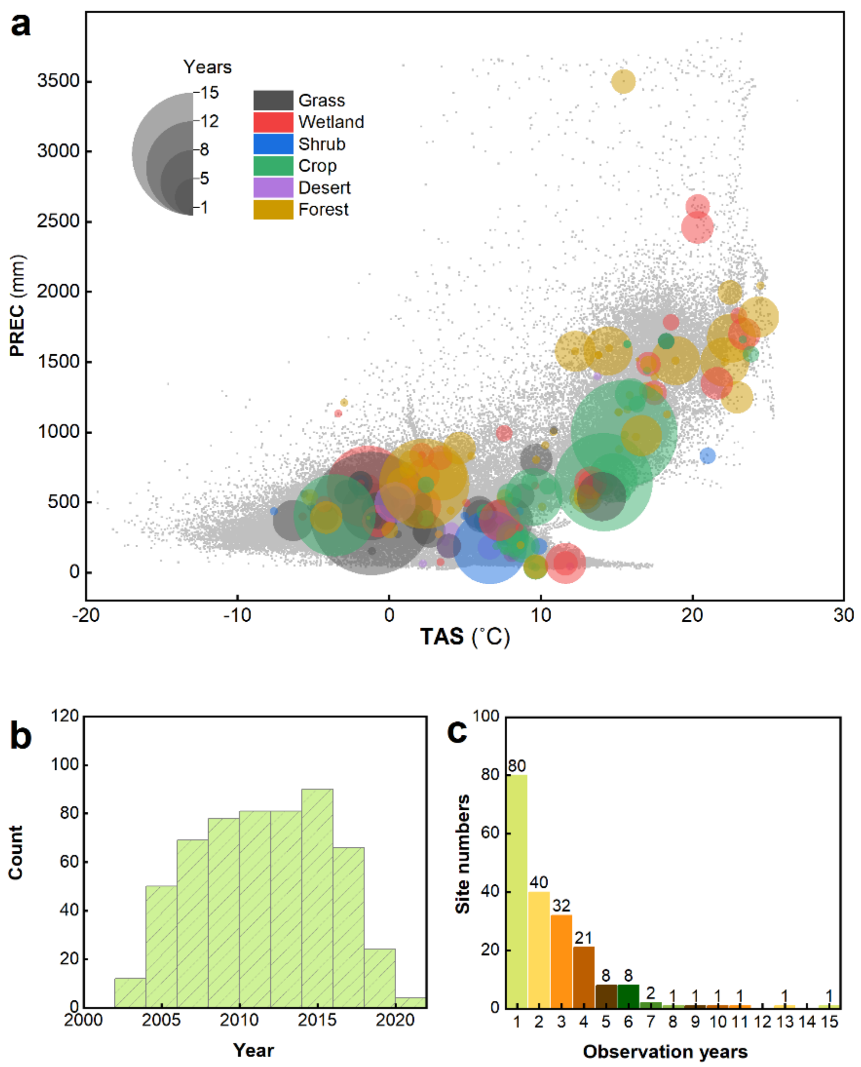

Fig. S1. Spatial and temporal features of the EddyChina2023 dataset. (a) Mean annual ambient temperature (TAS) and mean annual precipitation (PREC) of the eddy covariance sites in the EddyChina2023 dataset. The squares represent land surface pixels (0.1°×0.1°) in China and the colored dots indicate the eddy covariance sites in the EddyChina2023 dataset. The size of the circle represents the observation years for each site. **(b)** Histogram of the eddy covariance site observation years. **(c)** Duration of observations (in years) of the eddy covariance sites in the EddyChina2023 dataset.

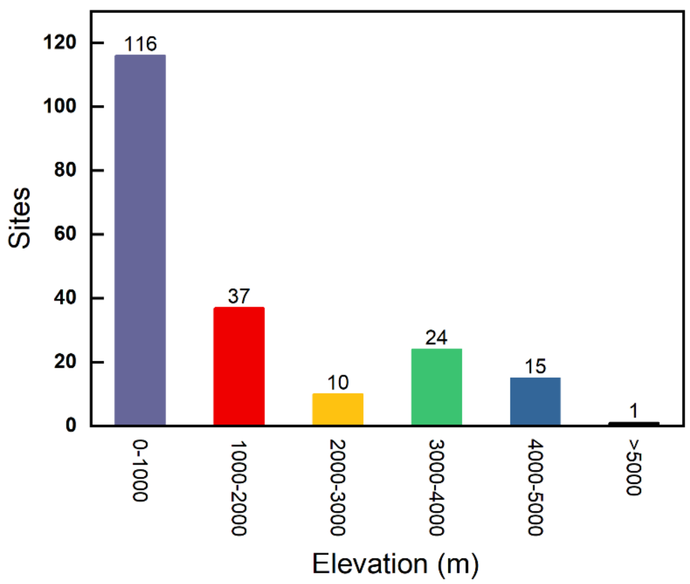

Fig. S2. Distribution of eddy covariance observation sites across elevation gradients. The numbers above each column represent the number of eddy covariance sites in each elevation band.

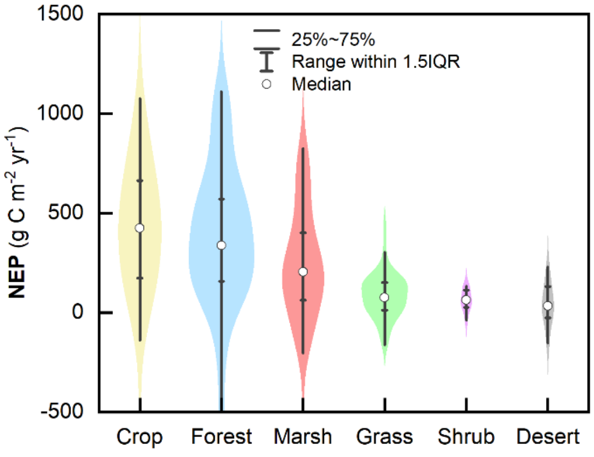

Fig. S3. Variation in the net ecosystem productivity (NEP) in various ecosystem types across China. The violin plots depict the magnitude and distribution density of the major vegetation types. Forest (n=61), grassland (n=43), wetland (n=38), cropland (n=41), desert (n=12), and shrubland (n=10)

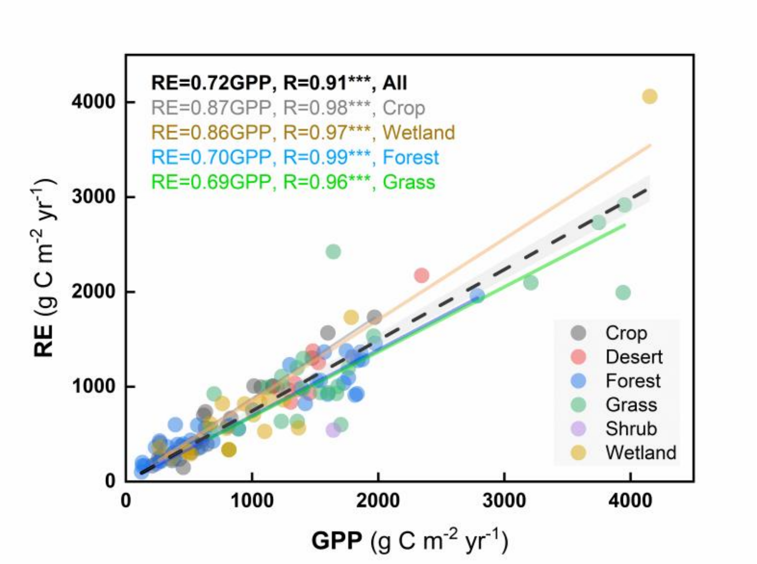

Fig. S4. Correlation between the gross primary productivity (GPP) and ecosystem respiration (RE) for terrestrial ecosystems in China. The dashed line indicates the linear fit of the RE for all sites (the central estimate) and the gray shading indicates the 95% confidence band of the linear fit. The colored lines indicate the linear fits for the major ecosystem types (cropland, wetland, forest and grassland); the linear fits are not given for desert and shrubland due to insufficient data. *** indicates P<0.001. Forest (n=61), grassland (n=43), wetland (n=38), cropland (n=41), desert (n=12), and shrubland (n=10)

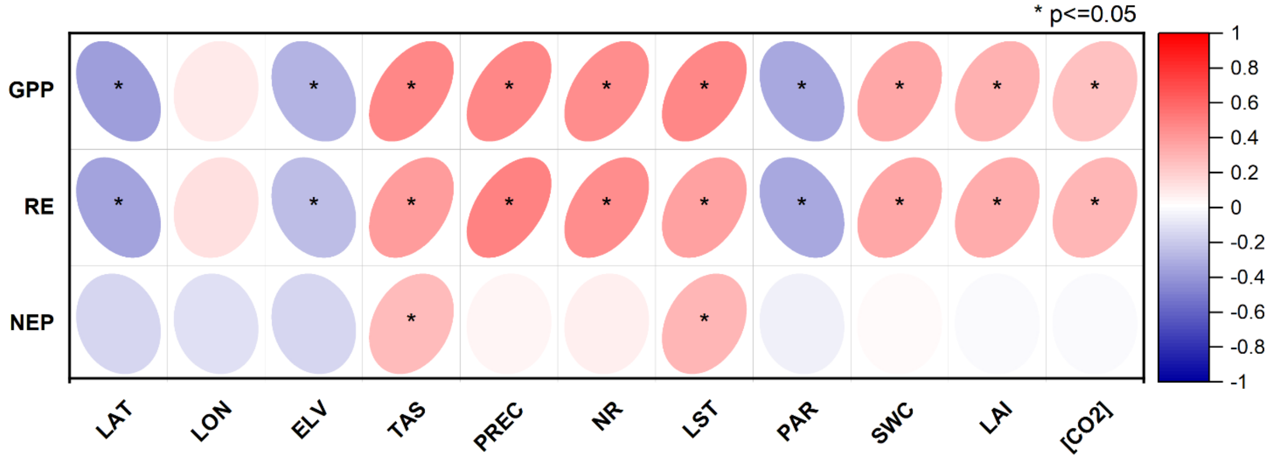

Fig. S5. Correlation relationships between gross primary productivity (GPP), ecosystem respiration (RE) and net ecosystem productivity (NEP) and climate, soil and permafrost factors. LAT, latitude; LON, longitude; ELV, elevation; TAS, atmospheric temperature; PREC, precipitation; NR, reactive N; LST, land surface temperature; PAR, photosynthetic active radiation; SWC, soil water content; LAI, leaf area index; [CO_2], atmospheric CO_2 concentration.

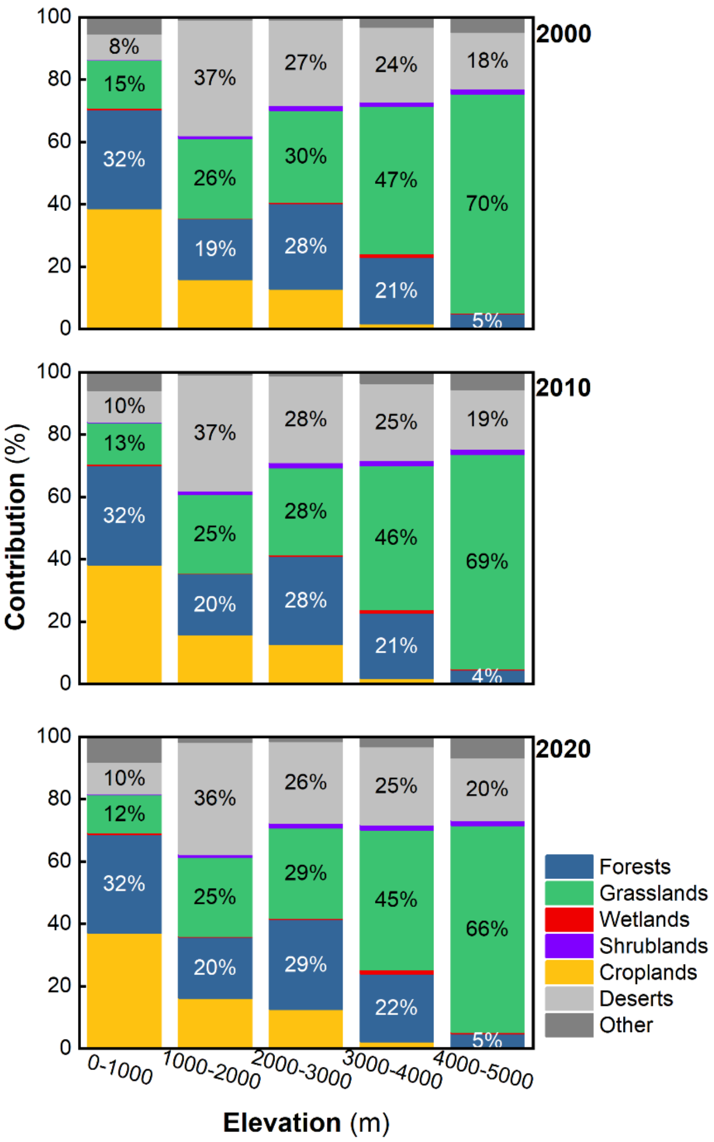

Fig. S6. Land cover and land use patterns across the elevation gradient in China. The land cover and use information was extracted from the 30-m resolution map of China (Globeland30) [1].

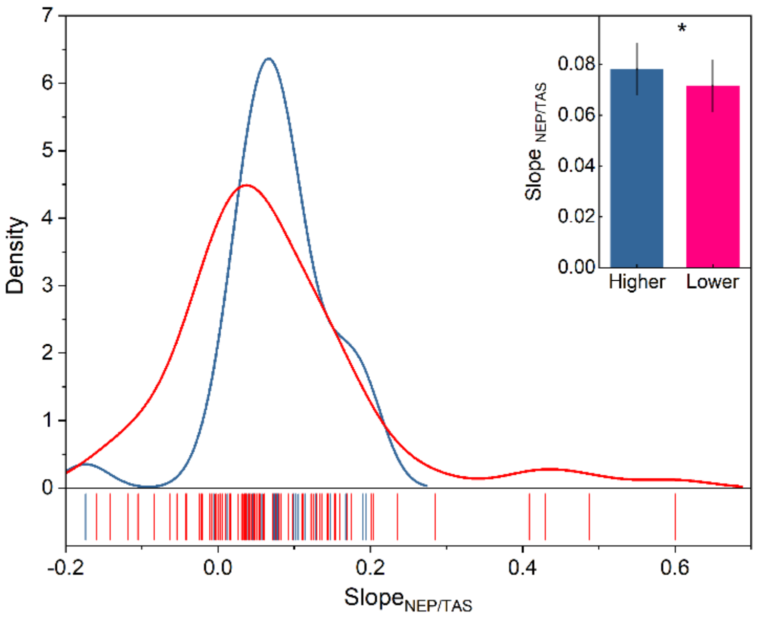

Fig. S7. Comparison of the temperature sensitivities of net ecosystem productivity between high-elevation environments and their lower elevation counterparts. High-elevation environments are defined as >3000 meter in elevation (denoted by "Higher"), while lower elevation environments as <3000 meters (denoted by "Lower"). The NEP sensitivity to temperature is defined as slope between NEP and temperature variation for each site ($Slope_{NEP/TAS}$; NEP, net ecosystem productivity; TAS=Atmospheric Temperature). Each site's temperature sensitivity was independently calculated. Then, the difference of temperature sensitivities between two groups, i.e., high- and lower elevation environments, were tested with Independent-Samples T test. The bar indicates standard deviation. The mean temperature sensitivity for high- and lower-elevation environments are 0.08±0.01 (n=33 sites) and 0.07±0.01 g C °C^{-1} (n=78 sites). The "*" indicates significant difference between two groups (*P*<0.05).

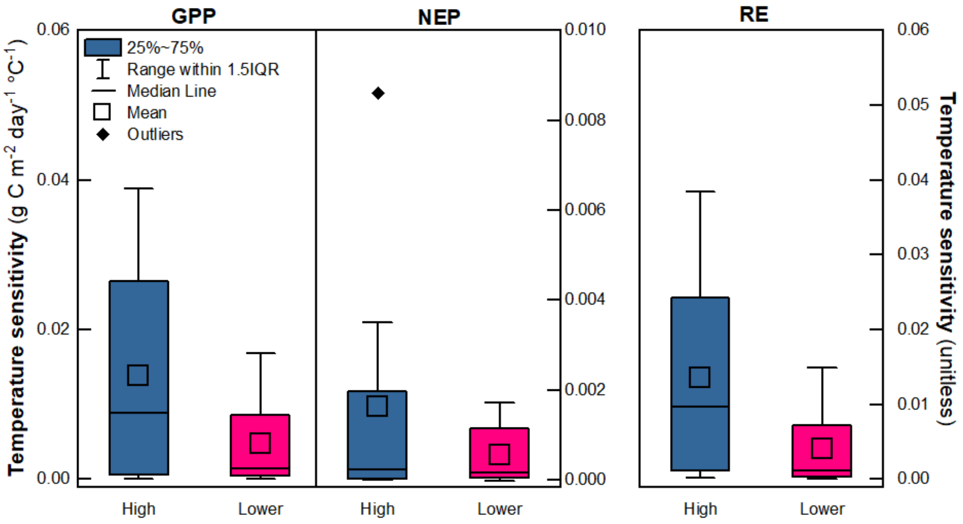

Fig. S8 Temperature sensitivity of gross primary productivity, ecosystem respiration and net ecosystem productivity based upon the MsTMIP ensemble [2]. High, high-elevation environments; Lower, lower elevation environments; GPP, gross primary productivity; RE, ecosystem respiration; NEP, net ecosystem productivity. The temperature sensitivity of GPP, RE and NEP of each pixel was calculated following the procedure descripted in Figure 3. Linear slopes were used for GPP and NEP, while the slope of RE were calculated after logarithm transformation. During the calculation, all pixels of each elevation band were employed to derive the slopes. Then, the results of these models were averaged to obtain the ensemble average and the standard deviation for each elevation band. The mean, median and variations (e.g., 25~75%) indicates the difference among various models of the MsTMIP BG1 group (driven by climate change, nitrogen deposition, atmospheric CO2 enrich and land use change). The MsTMIP BG1 group includes 8 models, i.e., BIOME-BGC, CLASS-CTEM-N, CLM4, CLM4VIC, DLEM, ISAM, TEM6 and TRIPLEX-GHG. n=8 for each box plot.

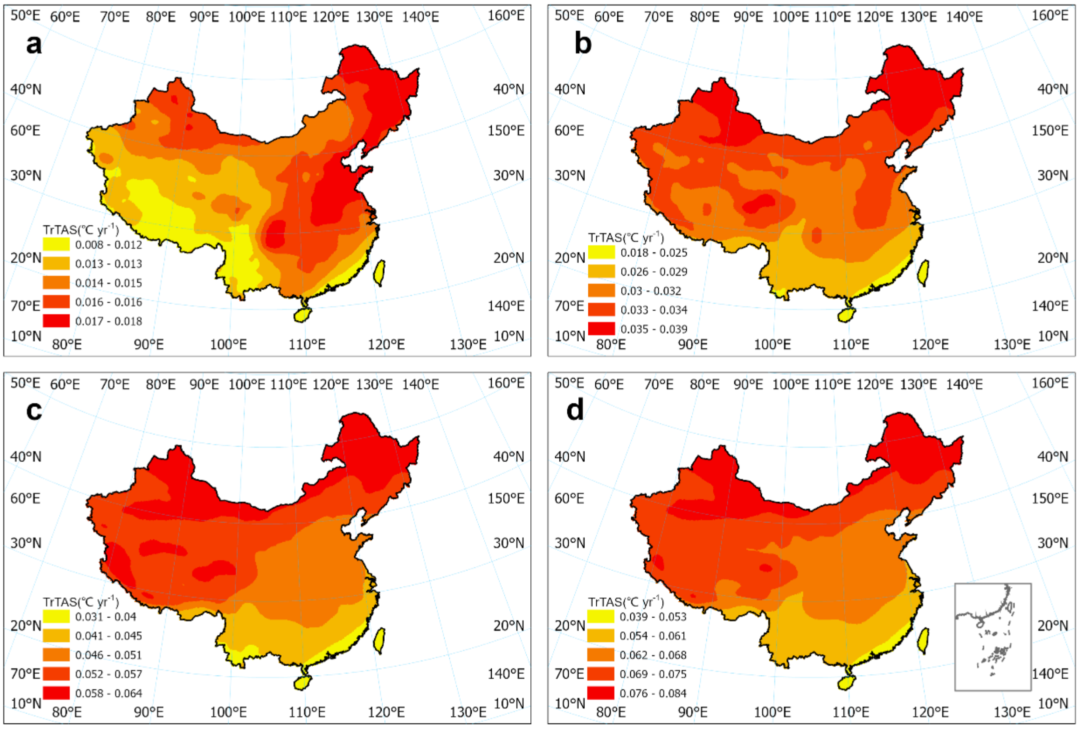

Fig. S9. Trend of the annual average air temperature under various climate scenarios. (a) SSP-1.26, (**b**) SSP-2.45, (**c**) SSP-3.70 and (**d**) SSP-5.85. TA, atmospheric temperature at a height of 2 m. Maps based on the outputs of Phase 6 of the Coupled Model Intercomparison Project (CMIP6) models [4].

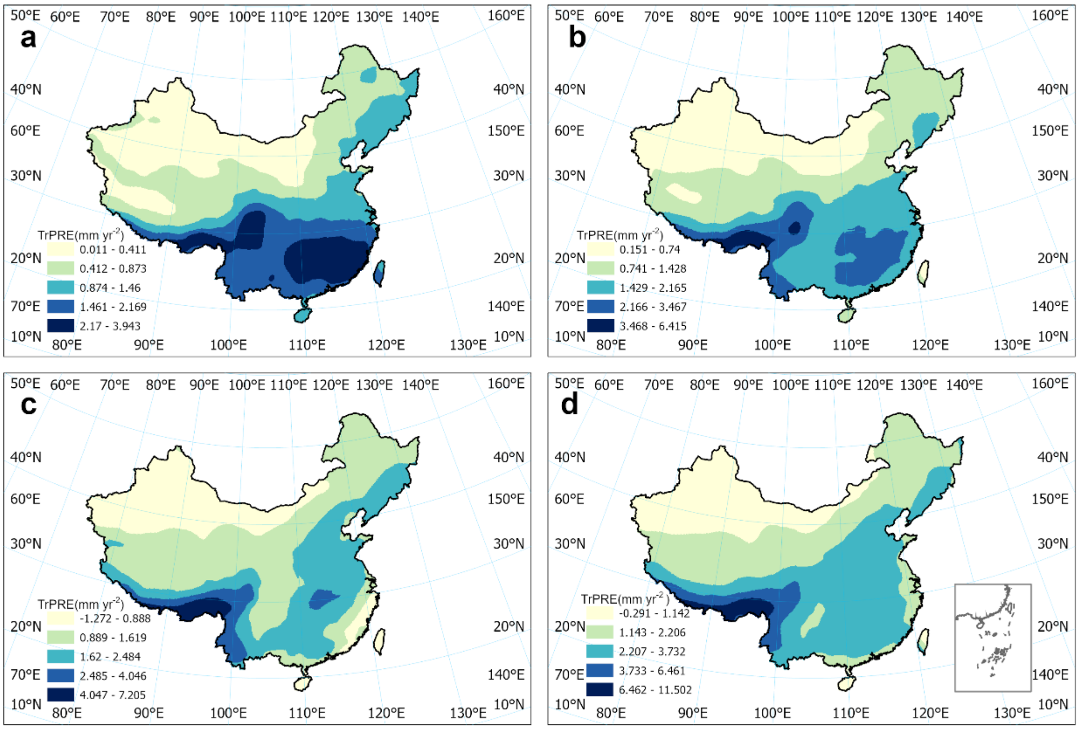

Fig. S10. Trend of annual precipitation under various Shared Socioeconomic Pathways. (a) SSP-1.26, (**b**) SSP-2.45, (**c**) SSP-3.70 and (**d**) SSP-5.85. PRE, precipitation. Maps based on the outputs of Phase 6 of the Coupled Model Intercomparison Project (CMIP6) models [4].

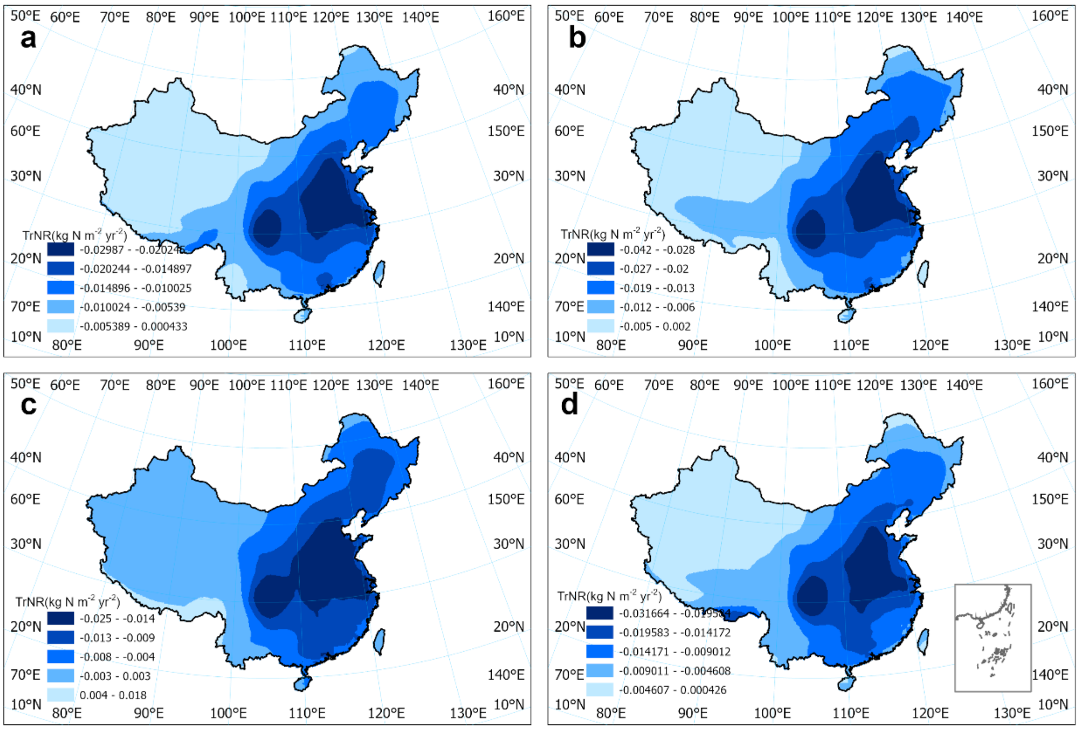

Fig. S11. Trend of the annual cumulative reactive N deposition under various shared socioeconomic pathways. **(a)** SSP-1.26, (**b**) SSP-2.45, (**c**) SSP-3.70 and (**d**) SSP-5.85. NR, reactive nitrogen. Maps based on the outputs of Phase 6 of the Coupled Model Intercomparison Project (CMIP6) models [4].

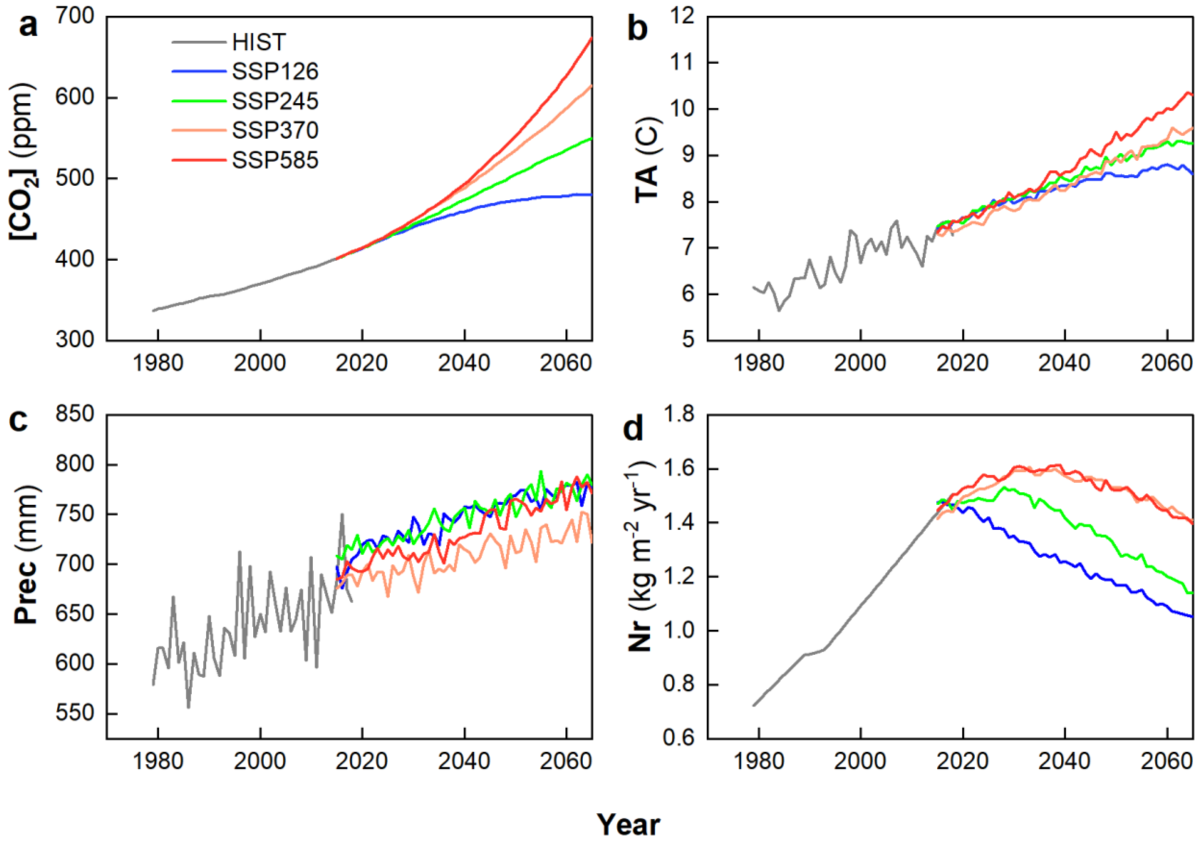

Fig. S12. Future variation in climate factors under various climate scenarios of the CMIP6 models. (a) Mean annual average atmospheric CO_2 concentrations across China. **(b)** Mean annual average atmospheric temperature at a height of 2 m across China. **(c)** Mean annual cumulative precipitation in China. **(d)** Mean annual average reactive nitrogen deposition in China. CMIP6, Phase 6 of the Coupled Model Intercomparison Project; [CO_2], atmospheric CO_2 concentration; TA, atmospheric temperature at a height of 2 m; Prec, precipitation; Nr, reactive nitrogen. Maps based on the outputs of Phase 6 of the Coupled Model Intercomparison Project (CMIP6) models [4].

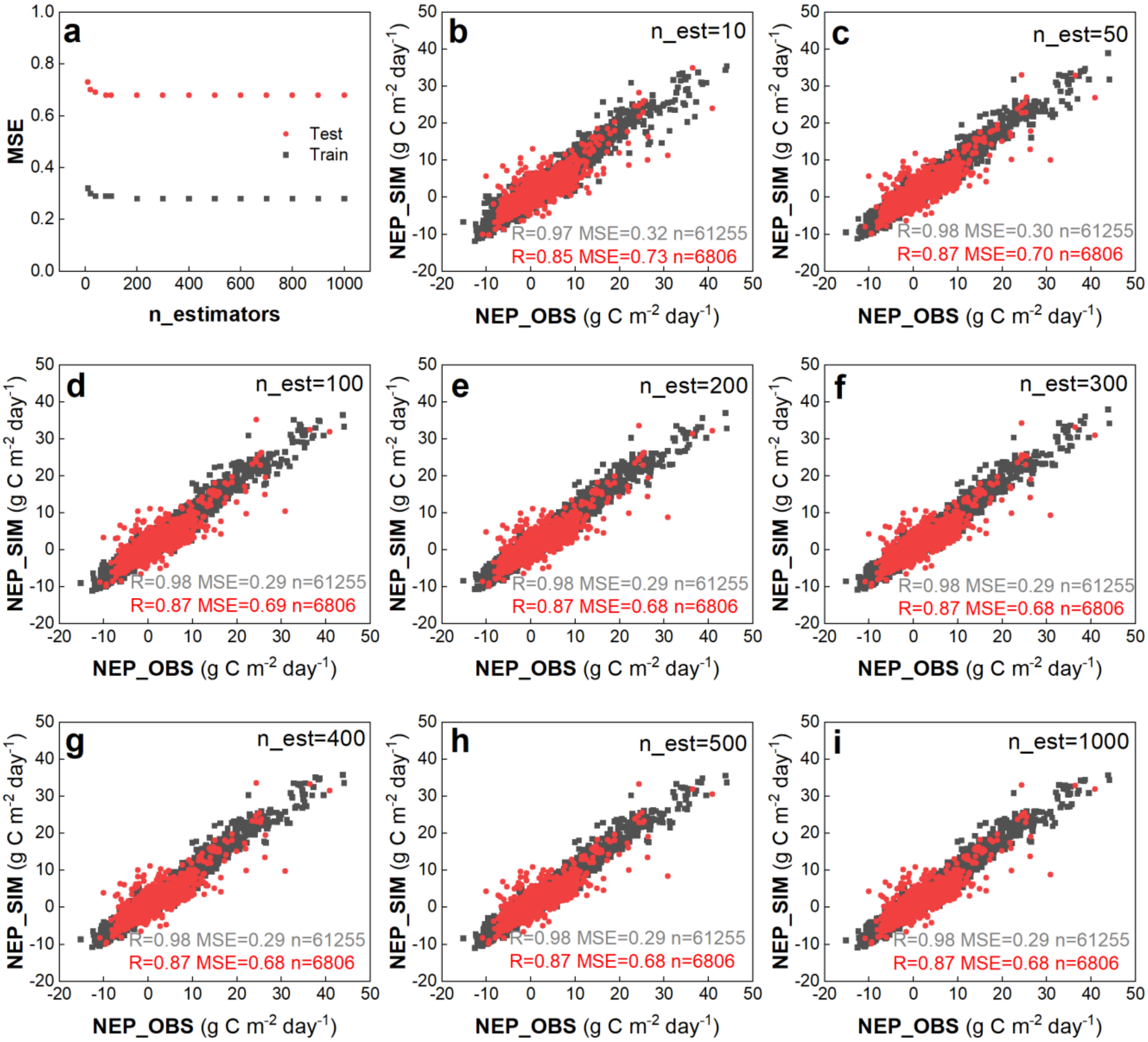

Fig. S13. Performance of the random forest algorithm in reproducing the net ecosystem productivity in China. **(a)** Variation in MSE with increasing number of estimators. (**b–i**) Correlation between the simulated and observed NEP across China's terrestrial ecosystems. NEP, net ecosystem productivity; MSE, mean square error; NEP_OBS, observed NEP; NEP_SIM, simulated NEP; n_est, number of estimators.

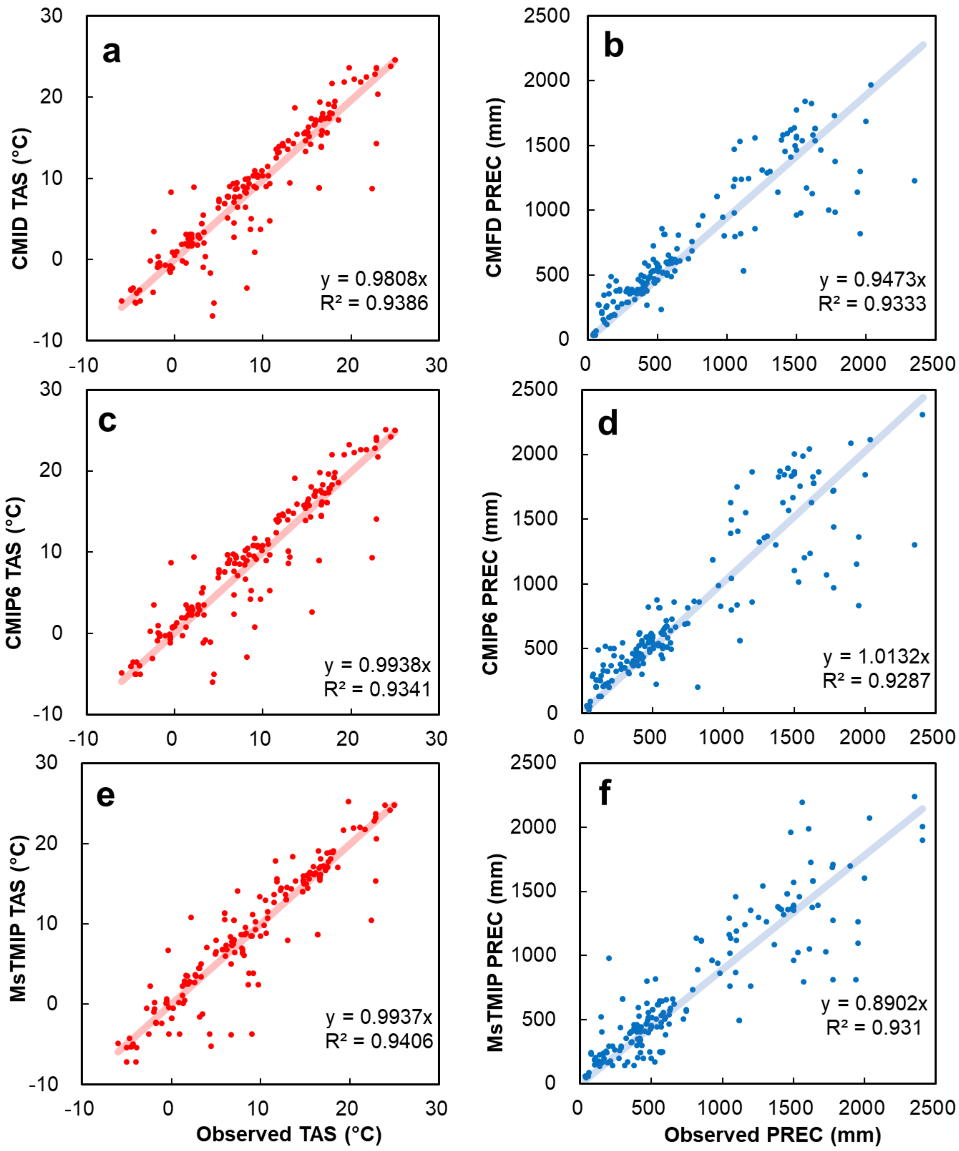

Fig. S14. Comparison among temperature/precipitation from ground-based observations, CMFD, WorldClim, CMIP6 datasets and MsTMIP climate datasets. (**a-b**) Correlation between ground-based observations and CMFD; (**c-d**) Correlation between ground-based observations and CMIP6; (**e-f**) Correlation between ground-based observations and MsTMIP forcing data (CRUNCEP). CMFD=China Meteorological Forcing Data; MsTMIP=Multi-Scale Synthesis and Terrestrial Model Intercomparison Project; CMIP6= Coupled Model Intercomparison Project Phase 6; TAS=Air temperature; PREC=Precipitation. n=203 for each figure. The lines indicate linear fit and central estimates.

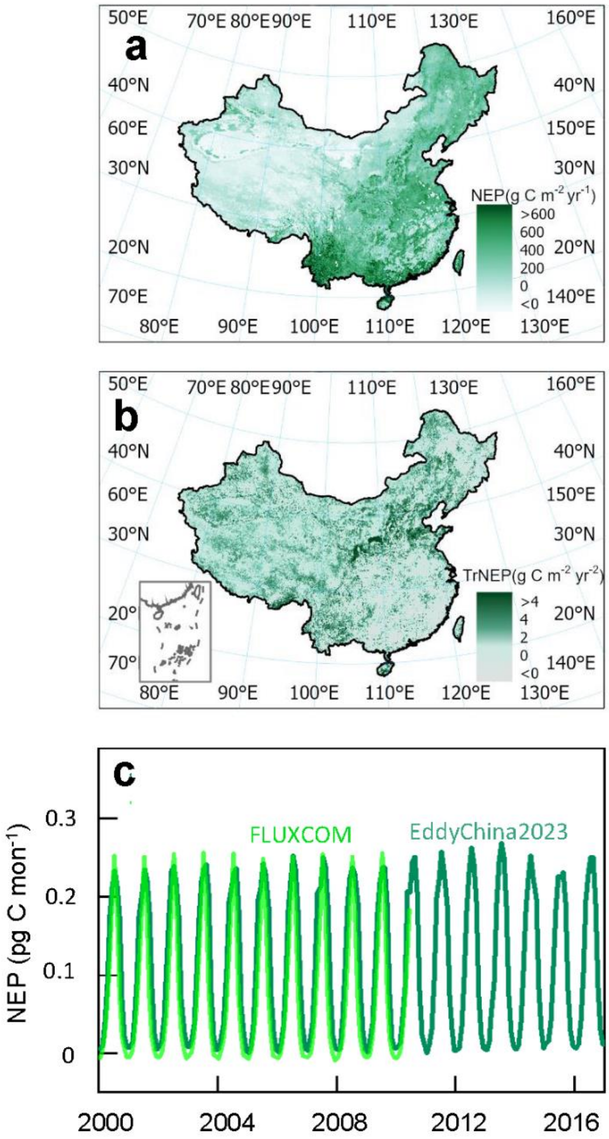

Fig. S15. Spatial pattern, trend, and magnitude of NEP across China's terrestrial ecosystems based upon the random forest training of EddyChina2023 dataset. (**a**) Averaged NEP of China's terrestrial ecosystems during the past two decades. (**b**) Trend of annual NEP of China's terrestrial ecosystems. (**c**) Comparison between monthly NEP of China's terrestrial ecosystems, estimated by EddyChina2023 and FLUXCOM [5]. The EddyChina2023-RF represents the results of random forest training based upon the EddyChina2023 dataset.

Table S1. List of eddy covariance observations across China's terrestrial ecosystems from the EddyChina2023 dataset.

Forest	Grassland	Wetland	Cropland	Desert	Shrub
Ailaoshan	Ali	Anqing	Changsha	Badain Jaran	Fukang
Anji (×2)	Ansai	Beihai	Changwu	Fukang	Haibei#3
Anqing	Arou	Changbaishan	Dali	Guazhou	Minqin
Badaling	Ban'ge	Dajiuhu	Daman	Gurbantunggut	Everest
Baotianman	Batang	Dangxiong	Dunhuang	Horqin	Shapotou
Changbaishan (×2)	Changling (×2)	Dashalong	Duolun-Crop	Huazhaizi	Shenmu
Changping	Dangxiong	Daxinganling	Fengqiu	Kubuqi	Tongliao
Danzhou	Duolun	Dongtan-High	Horqin	Ebinur	Yanchi
Daxing	Fenghuo	Dongtan-Low	Huailai	Siziwang	Yuanjiang
Daxing	Guoluo	Dongtan-Mid	Huining	Suqi	Yulin
Dinghushan	Guoluo (×2)	Dongying (×3)	Jinzhou	Taklimakan	
Dongguan	Guyuan	Gaoqiao	Kaidu-kongqi River	Zhangye	
Ebinur (×2)	Haibei	Haibei (×3)	Kuerle		
Ejin	Hailaer	Harbin	Kunshan		
Fengyangshan	Haiyan	Hongkong	Liuyang		
Gongga (×3)	Horqin	Hongyuan	Luancheng		
Guantan	Lijiang	Jiuduansha	Manasi		
Haidian Park	Maduo	Lake Poyang	Nanchang		
Hongzehu	Maqu	Lake Qinghai	Nanjing		
Huaining	Muztag	Leizhou	Poyang (×2)		
Huitong	Naiman	Panjin	Shihezi		
Huoditang	Nam Co	Sanjiang1 (×3)	Shiyanghe		
Huyanglin	Naqu (×2)	Shenzha	Shouxian (×2)		
Huzhong	Shenzha	Suganhu	Shouyang		
Jianfengling	Shuanghu	Taibei	Taoyuan		
Jinyunshan	Shulehe	Taihu	Tianshan		
Jurong	Suli	Taoyuan	Tongyu		
Lake Hongze	Sunid Zuoqi	Yueyang	Weishan		
Laoshan	Tanggula	Yunxiao	Wenjiang		
Liancheng	Tongyu	Zhangye	Wulanwusu		
Lijiang	Xilin (×2)	Zoige (×2)	Wuwei		
Linan (×3)	Xilinhot (×3)		Wuxi		
Linzhi	Yakou		Xuancheng		
Maoershan	Yuzhong		Xunxian		
Nantou	Zhangye		Yanting		
Ningxiang	Zoige		Yucheng		
Olympic Park			Yuncheng (×2)		
Puding			Zhangye		
Qianyanzhou					
Qidaoqiao					
Sidalong					
Songshan					
Taihe					
Tianmushan					
Wenchang					
Xianyang					
Xiaolangdi					
Xiping					
Banna (×2)					
Yichun					
Yueyang					
Zhangye					
Zhaoxian					

x2 indicates two eddy covariance towers; x3 indicates three eddy covariance towers.

Table S2. Models within the Multi-scale Synthesis and Terrestrial Model Intercomparison Project (MsTMIP) ensemble [2].

**Forcing data**	**Model**	**Resolution** (°Long. × °Lat.)	**Time period**
Climate forcing	BIOME-BGC	0.5×0.5	1901–2010
Land use	CLASS-CTEM-N	0.5×0.5	1901–2010
Atmospheric CO_2	CLM4	0.5×0.5	1901–2010
N deposition	CLM4VIC	0.5×0.5	1901–2010
	DLEM	0.5×0.5	1901–2010
	ISAM	0.5×0.5	1901–2010
	TEM6	0.5×0.5	1901–2010
	TRIPLEX-GHG	0.5×0.5	1901–2010

Long. = longitude; Lat. = latitude.

Table S3. Summary the Phase 6 of the Coupled Model Intercomparison Project (CMIP6) models used to drive the model prediction [4].

**SSP1-2.6**	**SSP2-4.5**	**SSP3-7.0**	**SSP5-5.8**
ACCESS-CM2	ACCESS-CM2	ACCESS-CM2	ACCESS-CM2
ACCESS-ESM1	ACCESS-ESM1	ACCESS-ESM1	ACCESS-ESM1-5
BCC-CSM2	BCC-ESM2	BCC-ESM2	BCC-CSM2-MR
CAMS-CSM1	CAMS-CSM1-0	CAMS	CAMS-CSM1
CanESM5	CanESM5	CanESM5	CanESM
CAS_ESM2	CAS-ESM2-0	CAS-ESM2-0	CAS-ESM2
CESM2	CESM	CESM2-WACCM	CESM2-WACCM
CMCC-CM2	CMCC-CM2-SR5	CMCC-CM2	CMCC-CM2
CMCC-ESM2	CMCC-ESM2	CMCC-ESM2	CMCC-ESM2
FGOALS-f3	FGOALS-f3-L	FGOALS-f3	FGOALS-f3
FGOALS-g3	FGOALS-g3	FGOALS-g3	FGOALS-g3
GFDL-ESM4	GFDL-ESM4	GFDL-ESM4	GFDL-ESM4
INM-CM4	INM-CM4	INM-CM4	INM-CM4
INM-CM5	INM-CM5	INM-CM5	INM-CM5
IPSL-CM6A	IPSL-CM6A	IPSL-CM6A	IPSL
KACE	KACE	KACE	KACE
MIROC6	MIROC6	MIROC6	MIROC6
MPI-ESM1-2-HR	MPI-ESM1-2-HR	MPI-ESM1-2-HR	MPI-ESM1-2-HR
MPI-ESM1-2-LR	MPI-ESM1-2-LR	MPI-ESM1-2-LR	MPI-ESM1-2-LR
NESM3	NESM3	TaiESM	NESM3
TaiESM	TaiESM		TaiESM

Supplementary References

1 Chen, J., *et al.* Open access to Earth land-cover map. *Nature* **514**, 434-434 (2014).

2 Huntzinger, D. et al. NACP MsTMIP: Global 0.5-deg Terrestrial Biosphere Model Outputs (version 1) in Standard Format, Data set. Data set. Available on-line [http:/daac.ornl.gov] from Oak Ridge National Laboratory Distributed Active Archive Center, Oak Ridge, Tennessee, USA, 10 (2018). https://doi.org/10.3334/ORNLDAAC/1225

3 He, J. *et al.* The first high-resolution meteorological forcing dataset for land process studies over China. *Scientific data* **7**, 25 (2020).

4 Eyring, V. *et al.* Overview of the Coupled Model Intercomparison Project Phase 6 (CMIP6) experimental design and organization. *Geosci. Model Dev.* **9**, 1937-1958 (2016).

5 Jung, M. *et al.* Scaling carbon fluxes from eddy covariance sites to globe: synthesis and evaluation of the FLUXCOM approach. *Biogeosciences* **17**, 1343-1365 (2020).

6 Tang, X. *et al.* Carbon pools in China's terrestrial ecosystems: New estimates based on an intensive field survey. *PNAS* **115**, 4021-4026 (2018).

7 Yang, Y. *et al.* Increased topsoil carbon stock across China's forests. *Global Change Biol.* **20**, 2687-2696 (2014).

8 Ding, J. *et al.* Decadal soil carbon accumulation across Tibetan permafrost regions. *Nat. Geosci.* **10**, 420-424 (2017).

9 Huang, Y. & Sun, W. J. Changes in topsoil organic carbon of croplands in mainland China over the last two decades. *Chin. Sci. Bull.* **51**, 1785-1803 (2006).

10 Zhu, X. J., *et al.* Research on the spatial-temporal variation of carbon consumption by agriculatural and forestry utilization in Chinese terrestrial ecosytems during 2000s. *Quaternary Sci.* **34**, 762-768 (2014).

11 Chen, C. *et al.* China and India lead in greening of the world through land-use management. *Nat. Sustain.* **2**, 122-129 (2019).

12 Yu, G. *et al.* High carbon dioxide uptake by subtropical forest ecosystems in the East Asian monsoon region. *PNAS* **111**, 4910-4915 (2014).

13 Tong, X. *et al.* Forest management in southern China generates short term extensive carbon sequestration. *Nat. Commun.* **11**, 10 (2020).

14 Yu, Z. *et al.* Mapping forest type and age in China's plantations. *Sci. Total Environ.* **744**, 140790 (2020).

15 Yang, Y. H. *et al.* Soil carbon stock and its changes in northern China's grasslands from 1980s to 2000s. *Global Change Biol.* **16**, 3036-3047 (2010).

16 Wei, D. *et al.* Plant uptake of CO_2 outpaces losses from permafrost and plant respiration on the Tibetan Plateau. *PNAS* **118**, e2015283118 (2021).
