# Supplementary material for: Elevation-dependent pattern of net CO2 uptake across China
Source: Nat Commun. 2024 Mar 20;15:2489. doi: 10.1038/s41467-024-46930-4 (PMC10954722; doi:10.1038/s41467-024-46930-4)
Supplement: Supplementary file 3 — Reporting Summary [file 41467_2024_46930_MOESM3_ESM.pdf]

nature portfolio

Corresponding author(s): Da Wei

Last updated by author(s): Mar 1, 2024

Reporting Summary

Nature Portfolio wishes to improve the reproducibility of the work that we publish. This form provides structure for consistency and transparency in reporting. For further information on Nature Portfolio policies, see our Editorial Policies and the Editorial Policy Checklist.

Statistics

For all statistical analyses, confirm that the following items are present in the figure legend, table legend, main text, or Methods section.

n/a	Confirmed	
☐	☒	The exact sample size (*n*) for each experimental group/condition, given as a discrete number and unit of measurement
☒	☐	A statement on whether measurements were taken from distinct samples or whether the same sample was measured repeatedly
☐	☒	The statistical test(s) used AND whether they are one- or two-sided<br>*Only common tests should be described solely by name; describe more complex techniques in the Methods section.*
☐	☒	A description of all covariates tested
☐	☒	A description of any assumptions or corrections, such as tests of normality and adjustment for multiple comparisons
☐	☒	A full description of the statistical parameters including central tendency (e.g. means) or other basic estimates (e.g. regression coefficient) AND variation (e.g. standard deviation) or associated estimates of uncertainty (e.g. confidence intervals)
☐	☒	For null hypothesis testing, the test statistic (e.g. *F*, *t*, *r*) with confidence intervals, effect sizes, degrees of freedom and *P* value noted<br>*Give P values as exact values whenever suitable.*
☒	☐	For Bayesian analysis, information on the choice of priors and Markov chain Monte Carlo settings
☒	☐	For hierarchical and complex designs, identification of the appropriate level for tests and full reporting of outcomes
☐	☒	Estimates of effect sizes (e.g. Cohen's *d*, Pearson's *r*), indicating how they were calculated

Our web collection on statistics for biologists contains articles on many of the points above.

Software and code

Policy information about availability of computer code

Data collection	EddyPro 7 (LI-COR, Lincoln, Lincoln, United States)
Data analysis	Random Forest Model in Python Environment (with netcdf4, scilearn, pandas and numpy packages)

For manuscripts utilizing custom algorithms or software that are central to the research but not yet described in published literature, software must be made available to editors and reviewers. We strongly encourage code deposition in a community repository (e.g. GitHub). See the Nature Portfolio guidelines for submitting code & software for further information.

Data

Policy information about availability of data

All manuscripts must include a data availability statement. This statement should provide the following information, where applicable:

- Accession codes, unique identifiers, or web links for publicly available datasets
- A description of any restrictions on data availability
- For clinical datasets or third party data, please ensure that the statement adheres to our policy

The manuscript include a data availability statement.
The EddyChina2023 data generated in this study have been deposited in the figureshae database under accession code https://figshare.com/s/e1f7f9c13e547e422a71. The MsTMIP data are available at https://daac.ornl.gov; the CMFD data are available from the National Tibetan Plateau Data Center

(http://data.tpdc.ac.cn); the CMIP6 climate data are from https://esgf-index1.ceda.ac.uk; the land-cover data are from https://modis.gsfc.nasa.gov; and the GLASS-GPP product is from http://www.geodata.cn/thematicView/GLASS.html.

Research involving human participants, their data, or biological material

Policy information about studies with human participants or human data. See also policy information about sex, gender (identity/presentation), and sexual orientation and race, ethnicity and racism.

Reporting on sex and gender	N/A
Reporting on race, ethnicity, or other socially relevant groupings	N/A
Population characteristics	N/A
Recruitment	N/A
Ethics oversight	N/A

Note that full information on the approval of the study protocol must also be provided in the manuscript.

Field-specific reporting

Please select the one below that is the best fit for your research. If you are not sure, read the appropriate sections before making your selection.

☐ Life sciences ☐ Behavioural & social sciences ☒ Ecological, evolutionary & environmental sciences

For a reference copy of the document with all sections, see nature.com/documents/nr-reporting-summary-flat.pdf

Ecological, evolutionary & environmental sciences study design

All studies must disclose on these points even when the disclosure is negative.

Study description	This study is achieved by collecting data (including observations by ourselves and literatures), and no experimental treatment is involved. In this study, we collected observations from 203 eddy covariance sites across China to determine the elevation-dependent pattern of NEP and its changes under a warming climate and changing human activities. We also explore the variation in NEP along the elevation gradient during the past four decades, based on biogeochemical models and satellite observations. Finally, we predict how the elevation-dependent pattern of NEP will be affected under various climate scenarios using machine learning, the eddy covariance dataset, and Coupled Model Intercomparison Project Phase 6 (CMIP6) models.
Research sample	All eddy covariance data obtained by our group has been incorporated into the EddyChina2023 (203 eddy covariance towers or 523 site-year).Several criteria were used to guarantee the quality of the data in the studies identified in the literature review. First, the data collection must have used the eddy covariance technique to ensure the spatial and temporal coverage. Observations based on static chambers, especially manual static chambers, were excluded because of their relatively small spatial coverage (typically <1 m2) and low temporal coverage (usually conducted in the daytime at weekly intervals). Second, the observations had to cover at least a whole year because CO2 emissions outside the growing season significantly affect the annual-scale CO2 sink. Third, the paper had to include a clear description of the eddy covariance installation, data collection and processing procedures (e.g., the Webb–Pearman–Leuning correction and axis rotation). All the sites were subjected to Webb–Pearman–Leuning density correction and were determined by the coordinate axis rotation approach—that is, 2D, 3D or planar fit coordinate axis rotation. The observational data had to be clearly and correctly presented, and papers with incorrect units of measurement were not included.
Sampling strategy	Raw data observed by our eddy covariance towers were processed by ourselves. Other data were obtained from peer-reviewed papers from the Web of Science (for English-language papers; http://apps.webofknowledge.com/) and the China National Knowledge Infrastructure (for Chinese papers; https://www.cnki.net/).
Data collection	For the raw data, the coauthors collectively collected these eddy covariance dataset from the field, including Da Wei, Jing Tao and Zhuangzhuang Wang. For the literature-based data, Jing Tao conducted the data collecting work. The following keywords were used during the literature search: eddy covariance; CO2 flux/exchange/sink/source; and China. From the perspective of the study period, most observations were conducted during 2002–2020, with a mean of 2012 From the perspective of climate coverage, the eddy covariance sites were well distributed over China's climate gradient, albeit with relatively fewer sites in cold–dry and hot–wet areas, which is consistent with the limited number of sites in northwestern China.
Timing and spatial scale	The data collection started from October 2020 to April 2021, covering the terrestrial ecosystem of China.
Data exclusions	The observational data had to be clearly and correctly presented, and papers with incorrect units of measurement were not included.
Reproducibility	We also employed process-based models and remote sensing dataset to validate the eddy covariance dataset. The models and remote sensing analyses both confirmed the results based upon the eddy covariance observations.

Randomization	All eddy covariance data available is collected to form a complete coverage of China's terrestrial ecosystems. The dataset was not allocated into experimental groups.
Blinding	To better cover the vast terrestrial ecosystems of China, we try to collect as much as possible data for machine learning. Furthermore, no experimental treatment was involved in this study, no "blinding" is needed for this study.

Did the study involve field work? ☒ Yes ☐ No

Field work, collection and transport

Field conditions	The field conditions of the eddy covariance towers on the Tibetan Plateau share a similar "dry and cold climate", i.e., annual mean temperature ranges from −6 to 8 °C, annual cumulative precipitation ranges from ~100 to ~600 mm per year.
Location	The field work cover lots of places in the Tibetan Plateau, including Maduo, Kekexili, Maidika, Yadong, Shenzha, Selincuo, Zhongba, Gaize and etc.
Access & import/export	We only collect the data generated by the eddy covariance towers (very much like data collection from auto weather station). All data collection completely comply with local law and no permission is needed for data collection.
Disturbance	Observations with Eddy covariance tower did not have any disturbance to local environments.

Reporting for specific materials, systems and methods

We require information from authors about some types of materials, experimental systems and methods used in many studies. Here, indicate whether each material, system or method listed is relevant to your study. If you are not sure if a list item applies to your research, read the appropriate section before selecting a response.

Materials & experimental systems

n/a	Involved in the study
☒	☐ Antibodies
☒	☐ Eukaryotic cell lines
☒	☐ Palaeontology and archaeology
☒	☐ Animals and other organisms
☒	☐ Clinical data
☒	☐ Dual use research of concern
☒	☐ Plants

Methods

n/a	Involved in the study
☒	☐ ChIP-seq
☒	☐ Flow cytometry
☒	☐ MRI-based neuroimaging

Plants

Seed stocks	N/A
Novel plant genotypes	N/A
Authentication	N/A
